# The Use of Pyoctanin in Treatment of Quittors

**Published:** 1895-09

**Authors:** Wilfried Lellmann

**Affiliations:** New York City


### THE USE OF PYOCTANIN IN THE TREATMENT OF QUITTORS.

BY DR. WILFRIED LELLMANN,
NEW YORK CITY.

ALTHOUGH the treatment described in this article has been followed by me in but a few cases, its invariable efficacy seems to me a sufficient warrant for its publication at this time.

The first thing I do is to apply a lukewarm foot-bath of a 1 per cent. solution of lysol. Then, after having examined the fistulous wound or wounds thoroughly with a probe, I introduce a bistoury, provided with a knob at its point, as deeply as possible and make a good cut as the bistoury is drawn outward. If only one fistula is present, I prefer to make a vertical and a horizontal cut, which meet at right-angles. If more fistulæ are present, I either connect them by cutting or make counter-openings, introducing drainage-tubes, but I must say that I prefer cutting. After this has been done I introduce a tampon of oakum, soaked with a 10 per cent. alcoholic solution of bichloride of mercury, put a well-pressing bandage on and leave it there for twenty-four hours. Now the fistulous wound has become large enough, and I commence at once with injections of a 10 per cent. alcoholic solution of pyoctanimum cæruleum. This is a pure aniline color, a methyl-violet, a blue scentless powder, which dissolves very easily in water and alcohol. It is non-poisonous, of great antiseptic power, and very siccative. Pyoctanin seems to have the peculiarity besides, to thrust off tissue which has undergone necrosis. After a few injections I am usually able to remove parts of the necrosed cartilage with forceps. Twelve to fifteen injections were sufficient; that during the first four or five days I made two injections daily, thereafter only one injection daily, but after each injection I tamponed the wound and applied a well-pressing bandage. After the injections have been applied I syringed the wound for several days with a solution of iodoform in ether, 1 : 7. After this treatment the wound or wounds became dry and did not show any more secretion. Now to accelerate the healing I introduced suppositories in the fistulous wounds and keep in position by a well-applied dressing.

The prescription of the suppositories I use is the following:

Bismuth. dithio. salicylic,
Butyr. cocæ et,
Lanolin. q. s. f. l. a. Bacilli suppositorii,
Longuitudine cm. crassitudine cm.

Bismuthum dithio-salicylicum, so-called thioform, is a new substitute for iodoform, and is greatly to be preferred to the latter. Thioform is a very light, fine and scentless powder of gray-yellowish color, insoluble in water. It is non-poisonous, and does not cause any irritation. In short, it seems to be superior to iodoform. When great swelling combined with a large defect of skin is present, as I saw it in several cases, I used bismuthum subgallicum, so-called "dermatol," with very good effect.

With the treatment above mentioned I had very good success. Although there was great lameness in all cases, the horses after this treatment could be put to work after two weeks and a half.

The shoes I prefer for horses with quittors, are plain, well-fitting, bar shoes, so as to remove weight and pressure from the seat of the injury. After the horses were put to work I left a good dressing, covered by a leather boot, for a week longer, so as to keep it clean and prevent interference with the injured part.

It is self-understood that in case a quittor is caused by pricks in shoeing or by suppurating corns, an opening must be made at the sole sufficiently wide to allow the free discharge of the contained pus.

I think, if the above-mentioned treatment is applied in a thorough way, it will be satisfactory in every point and will act even better and in shorter time than the operation, which can be performed only under certain circumstances, while this treatment seems to be feasible under any conditions.

I will not say that this treatment is the best; it may be that luck has been favorable to me during the treatment of the latest cases of quittors. The reason why I publish my treatment is the following:

For a great many veterinarians the treatment of quittors has been an ungrateful task, because the treatment lasted eight to ten weeks or even longer, often several months. By this time a great many horse-owners become impatient and they very often toward the end call on another veterinary surgeon, or, what is worse, they themselves commence to treat or consult a quack and he gets all the credit for it.

The cause of this certainly is to be seen in the continuous necrosis and suppuration of the cartilage. When we treat a fistulous wound in a right way, according to the principles of surgery—that is, when we cut the fistulous wound so as to change it into an incised wound, and allow free and fast escape of the contained pus, and remove the necrosed parts, which always keep up the suppuration and prevent the healing,—success most probably will show itself soon.

Now, I think that I have found in the 10 per cent. alcoholic solution of pyoctanimum cæruleum a remedy which I certainly will not claim to be a specific remedy, but which accelerates the thrusting off of necrosed tissue in a rapid way; besides, it is very destructive to staphylococci and streptococci, of siccative power, and seems to influence phlegmonous swellings in a very favorable way.

Although I have had good success in treating quittors with a 10 per cent. alcoholic solution of bichloride of mercury I prefer pyoctanin[1] because it is not dangerous. There are cases of quittors which I would not dare to treat with a 10 per cent. solution of bichloride of mercury, so highly recommended.

In conclusion, I would like to recommend this treatment to my colleagues, and I trust that they will find it very effective, if applied in the proper way.

[1] An objectionable feature in handling a solution of pyoctanin is its tendency to stain the fingers an intense blue, which, however, can easily be removed by "spiritus saponatus." I prefer to use a rubber glove while making injections.

